# Pleiotropic Effects of Simvastatin and Losartan in Preclinical Models of Post-Traumatic Elbow Contracture

**DOI:** 10.3389/fbioe.2022.803403

**Published:** 2022-02-21

**Authors:** Michael A. David, Alex J. Reiter, Chelsey L. Dunham, Ryan M. Castile, James A. Abraham, Leanne E. Iannucci, Ishani D. Shah, Necat Havlioglu, Aaron M. Chamberlain, Spencer P. Lake

**Affiliations:** ^1^ Department of Mechanical Engineering and Materials Science, Washington University in St. Louis, St. Louis, MO, United States; ^2^ Department of Biomedical Engineering, Washington University in St. Louis, St. Louis, MO, United States; ^3^ Department of Pathology, John Cochran VA Medical Center, St. Louis, MO, United States; ^4^ Department of Orthopaedic Surgery, Washington University in St. Louis, St. Louis, MO, United States

**Keywords:** post-traumatic joint contracture, rat elbow, gel contraction, simvastatin, losartan

## Abstract

Elbow trauma can lead to post-traumatic joint contracture (PTJC), which is characterized by loss of motion associated with capsule/ligament fibrosis and cartilage damage. Unfortunately, current therapies are often unsuccessful or cause complications. This study aimed to determine the effects of prophylactically administered simvastatin (SV) and losartan (LS) in two preclinical models of elbow PTJC: an *in vivo* elbow-specific rat injury model and an *in vitro* collagen gel contraction assay. The *in vivo* elbow rat (*n* = 3–10/group) injury model evaluated the effects of orally administered SV and LS at two dosing strategies [i.e., low dose/high frequency/short duration (D1) vs. high dose/low frequency/long duration (D2)] on post-mortem elbow range of motion (*via* biomechanical testing) as well as capsule fibrosis and cartilage damage (*via* histopathology). The *in vitro* gel contraction assay coupled with live/dead staining (*n* = 3–19/group) evaluated the effects of SV and LS at various concentrations (i.e., 1, 10, 100 µM) and durations (i.e., continuous, short, or delayed) on the contractibility and viability of fibroblasts/myofibroblasts [i.e., NIH3T3 fibroblasts with endogenous transforming growth factor-beta 1 (TGFβ1)]. *In vivo*, no drug strategy prevented elbow contracture biomechanically. Histologically, only SV-D2 modestly reduced capsule fibrosis but maintained elevated cellularity and tissue hypertrophy, and both SV strategies lessened cartilage damage. SV modest benefits were localized to the anterior region, not the posterior, of the joint. Neither LS strategy had meaningful benefits in capsule nor cartilage. *In vitro*, irrespective of the presence of TGFβ1, SV (≥10 μM) prevented gel contraction partly by decreasing cell viability (100 μM). In contrast, LS did not prevent gel contraction or affect cell viability. This study demonstrates that SV, but not LS, might be suitable prophylactic drug therapy in two preclinical models of elbow PTJC. Results provide initial insight to guide future preclinical studies aimed at preventing or mitigating elbow PTJC.

## 1 Introduction

Elbow trauma can lead to the debilitating condition of post-traumatic joint contracture (PTJC) in ∼8–50% of afflicted elbows (Anakwe et al., 2011; Myden and Hildebrand, 2011; Monument et al., 2013; Wessel et al., 2019; Hildebrand et al., 2021). An elbow affected by PTJC becomes contracted and stiff, leading to reduced range of motion and function (Anakwe et al., 2011; Myden and Hildebrand, 2011; Monument et al., 2013; Wessel et al., 2019; David et al., 2021; Hildebrand et al., 2021). Elbow PTJC is largely associated with capsule fibrosis, but can involve injury to other soft tissues like muscle, ligament, and cartilage (Monument et al., 2013; David et al., 2021; Hildebrand et al., 2021; Pooley and Van der Linden, 2021). When severe elbow PTJC develops, procedures removing the fibrotic capsule (i.e., capsulotomy) or treating arthritic cartilage (e.g., joint fusion or arthroplasty) serve as surgical options to improve forearm function (Monument et al., 2013; David et al., 2021; Hildebrand et al., 2021; Papadopoulos et al., 2021; Pooley and Van der Linden, 2021). However, the prognosis of these strategies is largely unpredictable, and these strategies are often unsuccessful in restoring full-motion/function and/or are associated with surgical revisions or complications (Monument et al., 2013; David et al., 2021; Hildebrand et al., 2021; Papadopoulos et al., 2021; Pooley and Van der Linden, 2021). Importantly, these approaches fail to address the biological underpinnings of PTJC, especially during the immediate-early biological response post-trauma (Monument et al., 2013; David et al., 2021; Hildebrand et al., 2021). Thus, novel disease-modifying strategies are needed to prevent or mitigate elbow PTJC.

Successful preventative treatments for elbow PTJC will likely depend on the disease severity at the time of intervention and the soft tissues (e.g., capsule and cartilage) responding to the aberrant biomechanical and biochemical stimuli post-trauma. Further, since these stimuli can cause joint-wide changes, it is important to consider the impact of injury and treatment on spatial changes in soft tissues at tissue and cellular levels. Similar to other fibrotic diseases, these aberrant stimuli are thought to drive a sustained increase in the number of myofibroblasts in the capsule that contract the tissue and deposit fibrotic matrix (e.g., collagen and proteoglycan), leading to fibrosis, stiffness, and loss of elbow motion (Monument et al., 2013; David et al., 2021; Hildebrand et al., 2021). In the cartilage, aberrant stimuli post-trauma might drive chondrocytes to overproduce proinflammatory, profibrotic, and catabolic factors, as well as activate chondrocyte proliferation and death (Zuscik et al., 2008; Anderson et al., 2011; Goldring, 2012; David et al., 2017). Consequentially, irreversible changes can occur to cartilage composition (e.g., loss of proteoglycan) and structure (e.g., surface fibrillation), leading to cartilage erosions (Zuscik et al., 2008; Anderson et al., 2011; Goldring, 2012; David et al., 2017). Taken together, preventative strategies modulating the immediate-to-early injury response of fibroblasts/myofibroblasts and chondrocytes in the capsule and cartilage, respectively, will reduce soft tissue damage and elbow contracture.

In the search for preventative treatments for elbow PTJC, two FDA-approved drugs, namely simvastatin (SV) and losartan (LS), offer potential options because of their pleiotropic effects in multiple organs and diseases. Classically, SV targets the mevalonate pathway, which is an essential pathway for cell health and metabolism (Stancu and Sima, 2001); whereas LS is an antagonist for the angiotensin II receptor type 1 (Bernasconi and Nyström, 2018), which modulates the renin-angiotensin physiologic system. These properties have led to the primary clinical use of SV and LS to treat hypercholesterolemia and hypertension, respectively, although these drugs have recently been considered for treating arthritis due to their potential ability to suppress inflammation in the joint capsule and synovial fluid, resulting in reduced cartilage damage (Cojocaru et al., 2013; Veronese et al., 2019; Wu et al., 2019, 2020). Additional preclinical studies show benefits of administration of both drugs in other diseases, including cartilage damage and joint swelling in the knee (Price et al., 2007; Yudoh and Karasawa, 2010; Aktas et al., 2011; Chen R. et al., 2015; Hamilton et al., 2018; Huard et al., 2018; Utsunomiya et al., 2020; Logan et al., 2021) and tissue fibrosis in the knee (Baranowski et al., 2019), lungs (Bagnato et al., 2013; Guo et al., 2015), muscle (Bedair et al., 2008; Burks et al., 2011; Kobayashi et al., 2013; Davis et al., 2015; Whitehead et al., 2015; Huard et al., 2018), and heart (Varo et al., 1999; Spurney et al., 2011; Sun et al., 2015; Böckmann et al., 2019; Kuo et al., 2019). Collectively, the aforementioned studies in the knee and other soft-tissues holistically suggest that both drugs might modulate fibroblasts/myofibroblasts and chondrocytes biology in the elbow joint post-trauma. Despite these pleiotropic benefits in other organ-tissues and diseases, both SV and LS’s impact on elbow PTJC remains unknown.

Therefore, this study aimed to evaluate the effects of SV and LS in two established preclinical models of contracture. Two SV and LS dosing strategies were tested in an elbow-specific rat injury model *in vivo*, which normally causes loss of elbow function, capsule fibrosis, and mild arthritis (Lake et al., 2016; Dunham et al., 2017a; Dunham et al., 2017b; Dunham et al., 2018a; Dunham et al., 2018b; Dunham et al., 2019; Dunham et al., 2020; Dunham et al., 2021; Reiter et al., 2019; Reiter et al., 2021a; Reiter et al., 2021b)*.* The *in vivo* model provides translatability of SV and LS therapy and joint-wide impact on multiple soft tissues (i.e., capsule and cartilage). Additionally, SV and LS were evaluated in a collagen gel contraction model *in vitro,* serving to mimic elbow capsule contraction *in vivo* (Hildebrand et al., 2014). The *in vitro* model allows for manipulation of experimental conditions on capsule cells of interest (i.e., fibroblasts/myofibroblasts). Overall, we hypothesized that SV and LS would prevent: 1) elbow contracture, capsule fibrosis, and cartilage damage *in vivo*; and 2) gel contraction of fibroblasts/myofibroblasts *in vitro*.

## 2 Methods

### 2.1 *In Vivo* Rat Elbow Injury Model

#### 2.1.1 Animals, Surgery, and Drug Strategies

**FIGURE 1:**
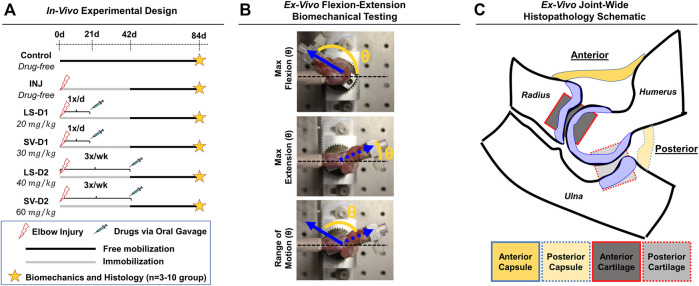
Experimental design for *in vivo* rat elbow injury model. **(A)** Summary of experimental groups, timeline, and post-mortem analysis. Control is uninjured, age-matched animals. For drug strategies, INJ is an injury with no treatment, while losartan (LS) and simvastatin (SV) represent injury plus LS and SV, respectively; dosage strategy 1 (D1) and dosing strategy 2 (D2) represents the treatment strategies of low dose/high frequency/short duration vs. high dose/low frequency/long duration, respectively. **(B)** Images depicting the *ex vivo* flexion-extension biomechanical testing set-up and the quantitative angular measurements obtained. In this elbow injury model, elbow contracture is characterized by i) decrease in the total range of motion, ii) increase in max extension angle (which translates into decreased elbow extension), and iii) unaltered max flexion. The total range of motion is calculated by determining the angle difference between max extension and max flexion. **(C)** Schematic of an elbow mid-sagittal section with the corresponding anatomical location of the capsule and cartilage for histopathology.

In this IACUC-approved study, male Long-Evans rats (*n* = 35; 330–370 g; Charles River Laboratories International, Wilmington, MA) were subjected to an established elbow injury model (Lake et al., 2016; Dunham et al., 2017b); male rats were selected because this PTJC model was developed using males and few sex-based differences have been observed (Reiter et al., 2021b). Briefly, unilateral elbows were subjected to anterior capsulotomy and transection of the lateral collateral ligament followed by a period of immobilization (0–42 days post-injury *via* bandage/wraps) and then free mobilization (i.e., unrestricted cage activity; 42–84 days post-injury) (Figure 1A). Immediately after injury, rats received either no drugs (INJ; *n* = 9) or one of two drug dosing strategies (D1 or D2; *n* = 3–5/group) administered *via* oral gavage: 1) LS-D1; 2) SV-D1; (iii); LS-D2; and 4) SV-D2. For D1, each drug was given 1x/daily for 3 weeks at 20 mg/kg and 30 mg/kg for LS and SV, respectively. For D2, each drug was given 3x/week for 6 weeks at 40 mg/kg and 60 mg/kg for LS and SV, respectively. Based on previous preclinical studies (Varo et al., 1999; Bedair et al., 2008; Aktas et al., 2011; Burks et al., 2011; Spurney et al., 2011; Bagnato et al., 2013; Kobayashi et al., 2013; Davis et al., 2015; Guo et al., 2015), the respective low and high doses were chosen, and the dosing strategies were categorized by either a low dose/high frequency/short duration (D1) or high dose/low frequency/long duration (D2); due to the difference in doses for each drug, no direct comparison between drug-treated groups is evaluated herein. Oral gavage was chosen as the delivery route to better control for the drug dose administered at a given time. Powdered forms of SV and LS were mixed with sterile water and adjusted for the weight of each rat. Elbows from uninjured, age-matched rats served as controls (Control; *n* = 10). After the free mobilization period, rats were humanely euthanized, and elbows were harvested for post-mortem biomechanical and histopathological analysis.

#### 2.1.2 Biomechanical Testing

Biomechanical analysis on *ex vivo* elbows from Control (*n* = 10), INJ (*n* = 9), D1 (*n* = 5/drug), and D2 (*n* = 3/drug) was performed as described previously (Lake et al., 2016; Dunham et al., 2017b; Reiter et al., 2019). Elbows were tested in flexion-extension to measure the maximum flexion, maximum extension, and range of motion (ROM), which collectively describe elbow motion (Figure 1B).

#### 2.1.3 Capsule and Cartilage Histological Semi-Quantitative Scoring

Following biomechanical testing, elbows (*n* = 3/group) were histologically processed for paraffin embedding, sectioned (mid-sagittal), and then stained with hematoxylin and eosin (H&E) and toluidine-blue (Tol-Blue) as previously performed (Lake et al., 2016; Dunham et al., 2017b, 2018a). Each section (*n* = 3 sections/stain/elbow) was blinded and semi-quantitatively scored by a musculoskeletal histopathologist (N.H.) using an adaptation of published metrics (Branchet-Gumila et al., 1999; Lake et al., 2016; Dar et al., 2017; Dunham et al., 2017b, 2018a). The semi-quantitative assessment of capsule and cartilage (Supplementary Table S1) included the characterization of cellular (e.g., cell number and type) and tissue (e.g., adhesions, fibrosis, thickness, and proteoglycans) level changes in both anterior and posterior anatomical joint regions (Figure 1C). For each histological section, a semi-quantitative metric was derived from a musculoskeletal histopathologist assessment. After evaluation, numerical scores for each elbow and group were averaged, converted into symbolic representation (−, +, ++, +++, or ++++), and then used for comparisons among groups.

#### 2.1.4 Cartilage Histomorphometry

Cartilage histomorphometry on the humerus was deployed using a method derived from previous techniques (Fukui et al., 2014; Dar et al., 2017; David et al., 2017). Briefly, sections (*n* = 1 representative section/elbow; chosen due to minimal slide to slide variation in histology scoring) were scanned under identical settings at 20x magnification (460 nm/pixel) using the NanoZoomer 2.0-HT System slide scanner (C9600-12; Hamamatsu, Shizuoka, Japan). Cartilage regions were defined in, and exported from, NanoZoomer Digital Pathology software (NDP-view2, Hamamatsu) and then processed through a semi-automatic script in MATLAB (Mathworks, Natick, MA). The following parameters were determined within the articular and calcified cartilages: 1) the number of chondrocytes; 2) the number of proteoglycan-rich chondrocytes (i.e., chondrocytes with intense pericellular Tol-Blue^+^ staining); 3) the number of empty lacunae; 4) the cartilage area; and 5) the proteoglycan-rich cartilage area (Tol-Blue^+^ staining). To obtain proteoglycan-rich cartilage, images were first color normalized to account for histological staining variation (i.e., scaled to each image’s white background and subchondral bone intensity) and then thresholded to remove non-proteoglycan rich pixels (based on average RGB pixel intensities for the subchondral bone). Proteoglycan amount was quantified by exploiting Tol-Blue’s metachromatic staining properties, where a darker Tol-Blue stain (i.e., lower average RGB pixel intensity) indicates more proteoglycan (Sridharan and Shankar, 2012).

### 2.2 *In Vitro* Gel Contraction and Live/Dead Assays

#### 2.2.1 Cell and Gel Culture

**FIGURE 2:**
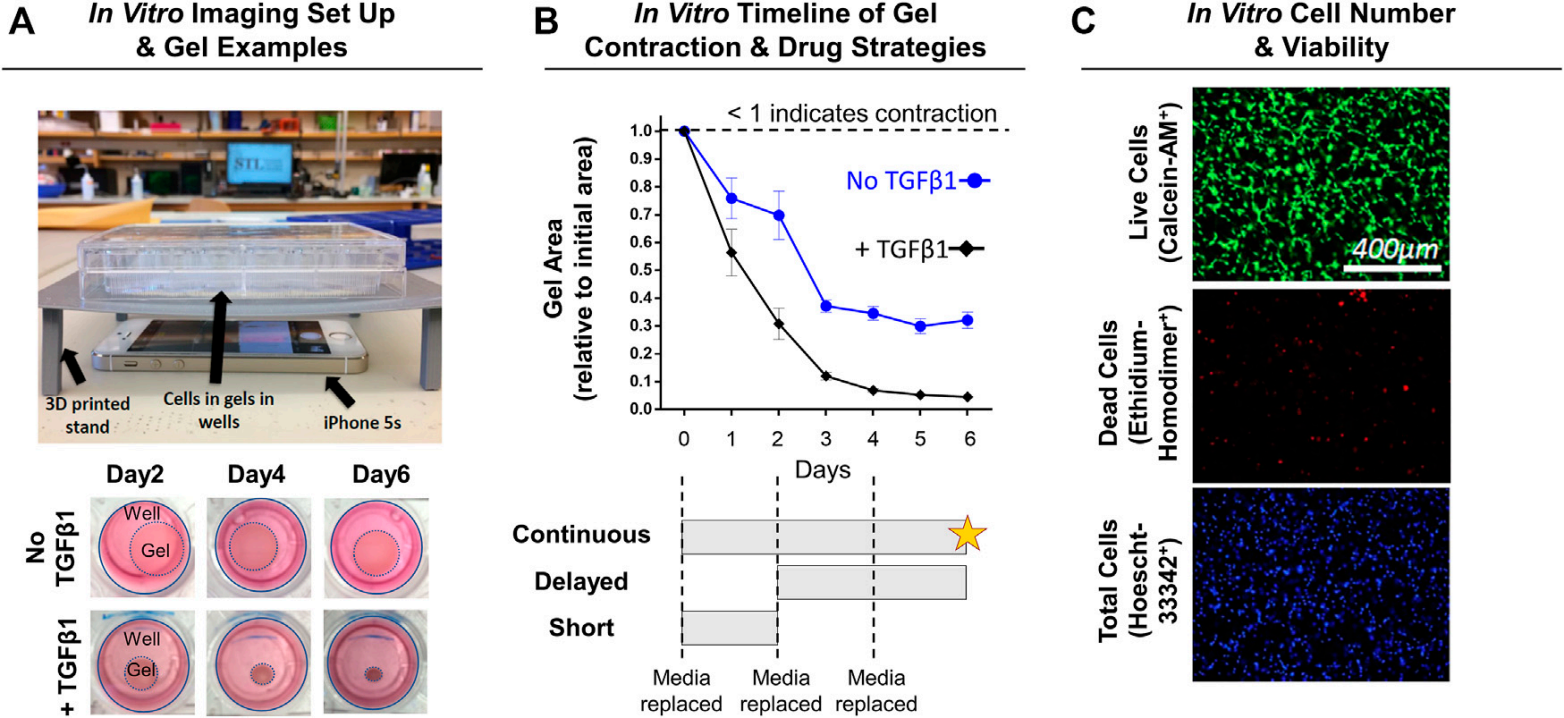
Experimental design for *in vitro* NIH3T3 fibroblast-embedded gel contraction. **(A)** Imaging set-up used to monitor gel contraction. Representative images of collagen gel (1.5 mg/ml) contraction by embedded NIH3T3 fibroblasts (5 × 10^5^ cells/ml) with and without TGFβ1 (10 ng/ml) over time. Note: Day 0 is not shown as the gels are approximately the same size as the well, and intermediate timepoints are sizes in between the days shown. **(B)** Quantification of gel contraction and drug dosing strategies used. Results are shown as average ± SEM (*n* = 12 for No TGFβ1; *n* = 19 for + TGFβ1). Note: star = live/dead assay. **(C)** Representative images of live, dead, and total cell staining used to quantify cell number (# of nuclei) and viability (% viable).

NIH3T3 fibroblasts (Sigma, St. Louis, MO) and transforming growth factor-beta one (TGFβ1; R&D Systems, Minneapolis, MN) were used as the cell line and profibrotic/contraction stimuli, respectively. This model system is routinely used to study the transdifferentiation of fibroblasts into myofibroblasts (Abdalla et al., 2013; Gutiérrez et al., 2015; Negmadjanov et al., 2015). Further, NIH3T3 fibroblasts were chosen in this study because primary capsule cells from rat or human tissue are not easily obtained, isolated, and expanded for high-throughput analysis. Briefly, NIH3T3 fibroblasts were cultured in media comprised of DMEM/High Glucose +10% fetal bovine serum and 1% penicillin-streptomycin (Fisher Scientific, Waltham, MA). Upon reaching ∼80% confluency, NIH3T3 fibroblasts were trypsinized and mixed into neutralized (pH 7; 300 mOsm) rat-tail collagen solution (collagen concentration of 1.5 mg/ml) at a density of 5 × 10^5^ cells/ml following previous methods (Cross et al., 2010; Iannucci et al., 2019). The collagen-cell mixture (500 µL) was then cast into uncoated 24-well plates (Midwest Scientific, Valley Park, MO) and polymerized for 1 h at 37°C before adding fresh media. After 24 h, gels were released from the wells using a sterile spatula to initiate spontaneous, free-floating gel contraction (Figure 2A). Immediately after releasing gels, SV or LS was added (1, 10, or 100 µM) with and without TGFβ1 (10 ng/ml) 1) every day (continuous), 2) for the first 2 days only (short), or 3) every day after a 2-days delay (delayed) (Figure 2B; *n* = 3–12 gels/group). These concentrations were chosen based on previous preclinical work and to test a range of concentrations several orders of magnitudes apart (Watson et al., 1998; Fürst et al., 2002; Porter et al., 2004; Watts et al., 2005; Benoit et al., 2008; Monzack et al., 2009; Burks et al., 2011; Copaja et al., 2011; Jia et al., 2016; Olschewski et al., 2018). Gels cultured in drug-free media with and without TGFβ1 served as controls (*n* = 12–19 gels/group). Culture media was changed every 2 days. Powdered SV, LS, and TGFβ1 were mixed into culture media following manufacture guidelines. A subset of gels (*n* = 2–3 gels/group) in drug-free media were supplemented with only the reconstitution solvents for SV (i.e., 0.001% dimethyl sulfoxide) and TGFβ1 (i.e., 0.002 mM hydrochloric acid) to verify that these solvents without drugs did not alter gel contraction.

#### 2.2.2 Collagen Gel Contraction Dynamics

Gels were imaged daily to monitor changes in gel contraction (i.e., gel area) by positioning an iPhone 5s (Apple, Cupertino, CA) below the culture plate resting on a custom-built stand (Figure 2A). Gel area was quantified using a custom MATLAB script, with results shown as a fraction of the initial gel area (Figure 2B).

#### 2.2.3 Cell Viability in Collagen Gels

After 6 days of continuous drug treatment with or without TGFβ1, gels (*n* = 4–11 gels/group) were stained with calcein-AM (2 μM; Fisher Scientific, Waltham, MA; FITC filter cube), ethidium homodimer-1 (4 μM; Fisher Scientific; TRITC filter cube), and Hoescht (2.5 μg/ml; Fisher Scientific; DAPI filter cube) dyes to identify live, dead, and total cells, respectively (Figure 2C). Gels were imaged within the gel interior (depths of ∼40 and ∼100 µm) at ×10 magnification (PlanFluor DLL 10x 0.30/16.00 mm; Nikon, Calgary, Canada) using an epifluorescent microscope (BZ-X810; Keyence, Itasca, IL). Cell number and viability at each gel depth was quantified and then averaged to obtain a representative gel value using a custom MATLAB script.

### 2.3 Statistics

Biomechanical data for INJ and Control were published previously and included for comparison (Reiter et al., 2019) but their histological sections were subjected to the analysis protocol herein (Dunham et al., 2017b). Statistical analysis was performed using GraphPad Prism 9 (GraphPad Software, San Diego, CA). For *in vivo* data, one-way ANOVA with Dunnett’s post-hoc test was performed to detect differences in biomechanical and cartilage histomorphometry parameters between INJ and drug-treated groups compared to Control. Of note, samples from LS-D2 were excluded from semi-quantitative scoring (*n* = 1) and histomorphometry (*n* = 2) because of histological processing errors and section folding. For *in vitro* data analysis, one-way ANOVA with Dunnett’s post-hoc test (against the respective drug-free condition) was performed for each parameter. Statistical significance was set at *p* ≤ 0.05, while trends were identified as 0.05 < *p* ≤ 0.10.

## 3 Results

### 3.1 *In Vivo* Rat Injury Model

#### 3.1.1 Rat Health and Elbow Biomechanics

**FIGURE 3:**
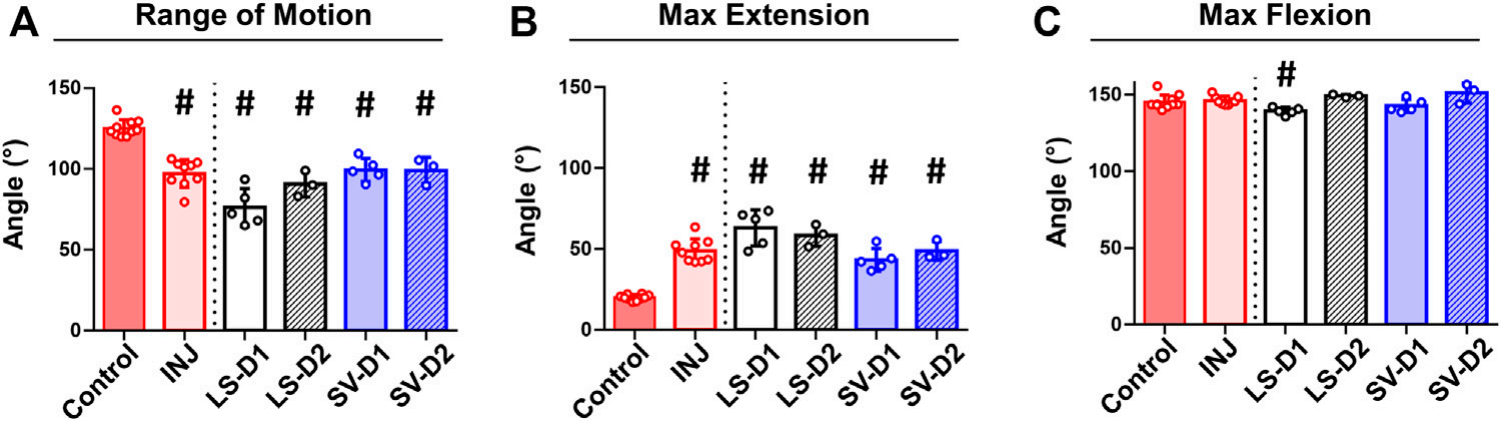
No drug strategy prevented biomechanical measures of elbow contracture. Biomechanical parameters of decreased range of motion **(A)** and increased max extension (i.e., decreased elbow extension) **(B)** demonstrate significant elbow contracture in all groups compared to Control; no treatment strategy prevented or improved elbow contracture. Max flexion **(C)** was largely unaffected by injury and treatment compared to Control. Results are shown as average ± SD; # indicates *p* < 0.05 significant difference from Control (one way-ANOVA with Dunnett’s post-hoc). Note: Control = uninjured, age-matched; INJ = injury no drug; LS-D1 = losartan dosing strategy 1; LS-D2 = losartan dosing strategy 2; SV-D1 = simvastatin dosing strategy 1; and SV-D2 = simvastatin dosing strategy 2.

No visible adverse side effects assessed by a veterinarian nor differences in rat weights among groups were observed (data not shown). Biomechanical testing revealed significantly decreased elbow range of motion (∼22%; Figure 3A) and extension motion (i.e., larger maximum extension values; ∼145%; Figure 3B) in INJ compared to Control. All drug strategies displayed similar changes in the range of motion and extension motion as in INJ compared to Control (Figures 3A,B). There were no differences in maximum flexion between any experimental group and Control except for LS-D1 (∼5%; Figure 3C). These biomechanics results indicate elbow contracture is not mitigated by drug treatments.

#### 3.1.2 Anterior and Posterior Capsule Histopathology

**FIGURE 4:**
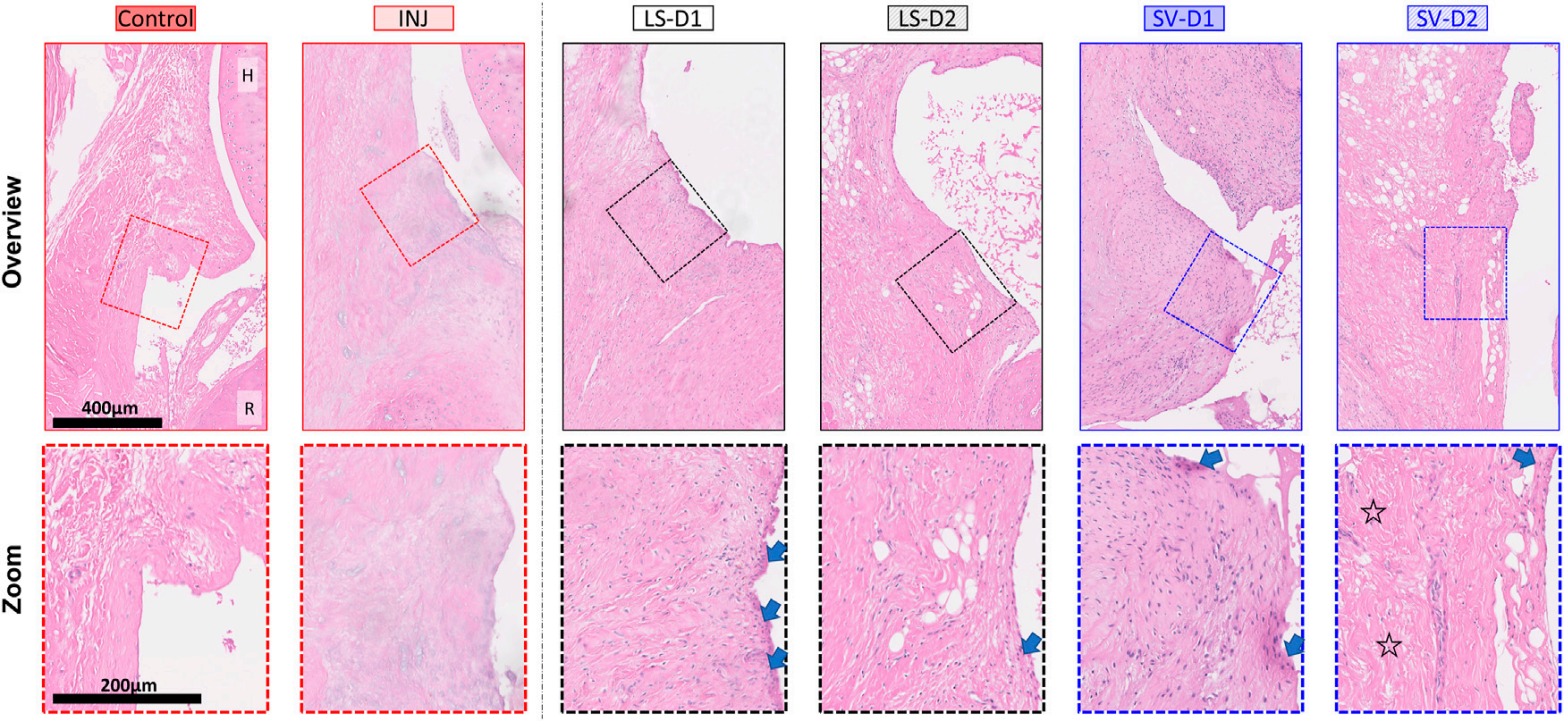
SV, but not LS, modestly reduced capsule fibrosis anteriorly, albeit with increased tissue thickness and cellularity*.* Qualitative histopathology assessment on H&E midsagittal sections of the anterior capsule highlights changes in the overall capsule tissue and cellular morphology. Control capsule displays loosely packed and disorganized tissue with minimal cellularity, whereas injury caused thickened tissue and fibrosis with minimal cells. Treatments displayed increased thickness, cellularity (mostly fibroblasts/myofibroblasts), synovial proliferation (arrows), and fibrosis. However, SV-D2 was the only group able to modestly reduce capsule fibrosis (open star). Note: H = humerus; R = radius; Control = uninjured, age-matched; INJ = injury no drug; LS-D1 = losartan dosing strategy 1; LS-D2 = losartan dosing strategy 2; SV-D1 = simvastatin dosing strategy 1; and SV-D2 = simvastatin dosing strategy 2.

Histopathology analysis revealed that all groups displayed different injury-induced responses at the tissue and cellular level in both the anterior (Table 1; Figure 4) and posterior (Table 1; Supplementary Figure S1) capsule. In the anterior capsule, INJ increased tissue-level parameters of capsule thickness, fibrosis, adhesion, and proteoglycans compared to Control (Table 1; Figure 4). With both LS treatments, similar tissue-level changes occurred in the INJ, except both resulted in a thickened, more fibrotic anterior capsule; LS-D2 also slightly reduced proteoglycans (Table 1; Figure 4). Similarly, both SV treatments had a different response to that observed in INJ (Table 1; Figure 4). SV-D2 slightly reduced fibrosis, proteoglycans, and adhesions compared to INJ, although these metrics were still elevated compared to Control (Table 1; Figure 4). There was no change in vascularity between groups (Table 1).

**TABLE 1:** Histological semi-quantitative scoring of the anterior and posterior capsule highlighted altered tissue and cellular properties post-injury and treatment. In the anterior capsule, INJ induced tissue-level thickening, proteoglycan deposition, and development of fibrosis and adhesions; SV-D2 is the only treatment that modestly reduced proteoglycans, adhesions, and fibrosis, albeit with increased tissue thickness, cellularity (predominantly fibroblasts/myofibroblasts), and synovial proliferation. Similar observations were made in the posterior capsule; however, SV-D2 no longer had capsular benefits in reducing tissue fibrosis, adhesions, or proteoglycans. In both anterior and posterior capsules, there was no change in vascularity or the number of mast cells and mononuclear inflammatory cells in any group compared to Control. Note: Histological parameters were semi-quantitatively assessed and given a symbol of either −, +, ++, +++, or ++++, where increases in the number of symbols (+vs. ++++) indicate worse disease severity; details on grading scheme is found in Supplementary Table S1; Control = uninjured, age-matched; INJ = injury no drug; LS-D1 = losartan dosing strategy 1; LS-D2 = losartan dosing strategy 2; SV-D1 = simvastatin dosing strategy 1; and SV-D2 = simvastatin dosing strategy 2.

Level	Parameter	Anterior Capsule							Posterior Capsule					
		Control	INJ	LS-D1	LS-D2	SV-D1	SV-D2		Control	INJ	LS-D1	LS-D2	SV-D1	SV-D2
Tissue	Thickness	−	++	++++	+++	++	++		+	+++	+++	++++	++++	++++
	Adhesions	−	++	++	++	++	+		−	++	+++	+++	+++	+++
	Fibrosis	−	++	+++	++	++	+		−	++	++	++	+++	+++
	Proteoglycan Amount	−	+	+	−	+	−		−	+	+	++	+	+
	Vascularity	+	+	+	+	+	+		+	+	+	+	+	+
Cellular	Cellularity	+	+	+++	++	+++	++		+	+	+++	++	+++	++
	Synovial Proliferation	−	−	+	+	+	+		−	+	+	+	+	+
	Fibroblasts/myofibrobasts	+	+	+++	++	+++	++		+	+	++	+	++	++
	Mast Cells	+	+	+	+	+	+		+	+	+	+	+	+
	Mononuclear Inflammatory Cells	+	+	+	+	+	+		+	+	+	+	+	+

At the cellular level of the anterior capsule, no differences were observed in total cellularity, synovial proliferation, and types of cells (fibroblasts/myofibroblasts, mast cells, or mononuclear inflammatory) in INJ compared to Control (Table 1; Figure 4). In contrast, every drug strategy led to increased cellularity, fibroblasts/myofibroblasts, and synovial proliferation compared to Control (Table 1; Figure 4). No drug strategy resulted in changes to mast or mononuclear inflammatory cells at the time point evaluated (Table 1).

For the posterior capsule, every drug strategy exhibited similar trends but increased scores at the tissue and cellular level compared to the anterior capsule (Table 1; Supplementary Figure S1). Notably, SV-D2 did not reduce fibrosis and adhesions in the posterior capsule as was seen in the anterior capsule (Table 1; Supplementary Figure S1). Collectively, these results indicate that only SV had modest benefits in capsule pathology (i.e., decreased fibrosis, adhesions, and thickening) through modulating the population of fibroblasts/myofibroblasts in the anterior region and not the posterior region.

#### 3.1.3 Anterior and Posterior Cartilage Histopathology

**FIGURE 5:**
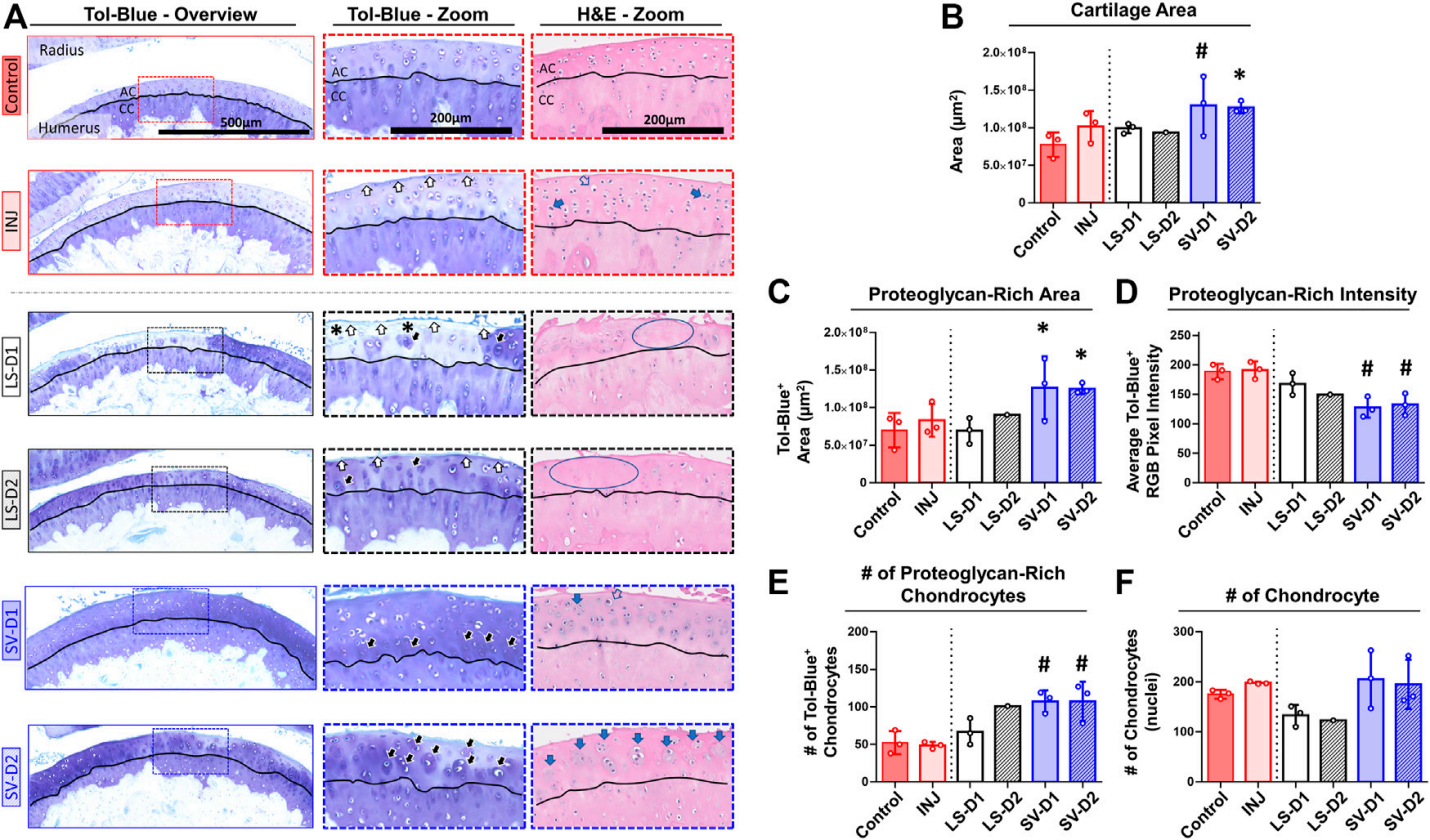
SV, but not LS, reduced cartilage surface irregularities and enhanced proteoglycan content anteriorly. **(A)** Qualitative histopathology assessment on toluidine blue (Tol-Blue) and hematoxylin and eosin (H&E) midsagittal sections of anterior humerus articular cartilage (AC) and calcified cartilage (CC) demonstrate minor cartilage surface-level fibrillations (white-filled arrow) with injury alone (INJ) compared to Control. Treatment with either LS strategy caused worse visible structural and modest proteoglycan loss (asterisks) changes. In contrast, both SV treatments appeared to reduce the severity of this damage and increased cartilage thickness, proteoglycan content, and proteoglycan-rich chondrocytes (black-filled arrow), as seen in Tol-Blue images. Note the minor loss of chondrocytes (blue circle) and the drastic change in chondrocyte morphology with an enlargement (open arrow) and cloning/clustering (blue-filled arrow) of chondrocytes in the articular cartilage, as seen in H&E images. **(B–F)** Quantitative cartilage histomorphometry of the humerus articular cartilage at the tissue level largely confirms these qualitative assessments while providing additional insight into cellular level changes in the number of chondrocytes and those with enriched pericellular proteoglycan. Results are shown as mean ± SD; # indicates *p* ≤ 0.05 significant difference and * indicates 0.05 < *p* ≤ 0.10 trending significance from Control (one-way ANOVA with Dunnett’s post-hoc). Note: Control = uninjured, age-matched; INJ = injury no drug; LS-D1 = losartan dosing strategy 1; LS-D2 = losartan dosing strategy 2; SV-D1 = simvastatin dosing strategy 1; and SV-D2 = simvastatin dosing strategy 2.

Histopathology assessment of the cartilage highlighted drastic changes at the tissue and cellular level depending on the anatomical location and the drug strategy. In the anterior region of INJ compared to Control, qualitative (Figure 5A) and semi-quantitative scores (Table 2) revealed mild cartilage damage in the form of surface irregularities, but without either a loss of proteoglycans, compromise in the tidemark integrity, or cellular morphological changes. Both LS strategies had a similar level of cartilage damage as INJ, except there was an additional loss of proteoglycans (Table 2; Figure 5A). On the contrary, SV-treated groups showed modestly reduced cartilage damage (Table 2; Figure 5A). While semi-quantitative metrics indicated no drastic changes in chondrocyte cellularity across entire cartilage (Table 2), qualitatively it appeared that injury with or without drug treatments induced subtle yet notable localized cellular morphology changes in the articular cartilage of the humerus (Figure 5A). Chondrocyte hypertrophy and cloning/clustering was observed in localized regions in INJ compared to Control (Figure 5A). For treatments, chondrocytes were somewhat absent in both LS groups while being enlarged and displaying clustering/cloning in both SV strategies (Figure 5A). Histomorphometry of the humerus cartilage revealed that INJ did not alter any parameter compared to Control (Figures 5B–F). However, SV strategies largely increased the overall cartilage area (Figure 5B), the distribution (i.e., area; Figure 5C) and amount (i.e., staining intensity; Figure 5D) of proteoglycan, and the number of proteoglycan-rich chondrocytes (Figure 5E). In contrast, LS strategies exerted no appreciable changes. For all groups, no appreciable changes in humerus cartilage histomorphometry were seen in the number of chondrocytes (Figure 5F) and empty lacunae (data not shown) in the articular cartilage or in any metric in the calcified cartilage (data not shown).

**TABLE 2:** Histological semi-quantitative scoring of the anterior and posterior cartilage demonstrated altered tissue and cellular properties post-injury and treatment. In both anterior and posterior cartilage, INJ caused minor surface irregularities compared to Control. In the anterior cartilage, both SV strategies could slightly prevent these minor surface irregularities, whereas LS had no benefit and even caused a loss of proteoglycan matrix staining. Posteriorly, no strategy provided cartilage protection; in fact, SV strategies worsened the severity of cartilage damage. Note: Histological parameters were semi-quantitatively assessed and given a symbol of either −, +, ++, +++, or ++++, where increases in the number of symbols (+vs. ++++) indicate worse disease severity; details on grading scheme is found in Supplementary Table S1; Control = uninjured, age-matched; INJ = injury no drug; LS-D1 = losartan dosing strategy 1; LS-D2 = losartan dosing strategy 2; SV-D1 = simvastatin dosing strategy 1; and SV-D2 = simvastatin dosing strategy 2.

Level	Parameter	Anterior Cartilage							Posterior Cartilage					
		Control	INJ	LS-D1	LS-D2	SV-D1	SV-D2		Control	INJ	LS-D1	LS-D2	SV-D1	SV-D2
Tissue	Structural Damage	−	+	+	+	−	−		−	+	+	+	+	++
	Proteoglycan Loss	−	−	+	+	−	−		−	+	+	+	+	+
	Tidemark Integrity	−	−	−	−	−	−		−	−	−	−	−	−
Cellular	Cellularity	−	−	−	−	−	−		−	−	−	+	−	−

Histopathology assessment of the posterior cartilage revealed mostly similar observations to the anterior cartilage. Qualitative (Supplementary Figure S2A) and semi-quantitative analyses (Table 2) showed posterior cartilage damage in INJ and both LS treatment groups compared to Control, including surface fibrillations and loss of proteoglycans; additional diffuse hypercellularity was seen in the LS-D2 group. Contrary to semi-quantitative observations in the anterior cartilage, both SV strategies caused significant cartilage erosions and loss of proteoglycan in the posterior region (Table 2). Qualitative assessment did not reveal striking changes in chondrocyte morphology in most groups, except the slight appearance of empty lacunae with both SV strategies (Supplementary Figure S2A). Articular cartilage histomorphometry (Supplementary Figures S2B–F) largely confirmed these qualitative and semi-quantitative observations with no changes in any parameter evaluated; however, despite erosions and loss of proteoglycan observed qualitatively and semi-quantitatively, there was no overall change in cartilage area or proteoglycans quantitatively. No appreciable cellular and tissue-level cartilage histomorphometry changes were observed in the posterior calcified cartilage (data not shown). Taken together, these results indicate that only SV had modest cartilage protection anteriorly through changes in chondrocyte appearance and extracellular matrix of the articular cartilage and not calcified cartilage.

### 3.2 *In Vitro* Gel Contraction

#### 3.2.1 Fibroblasts/Myofibroblasts Cell Contractility and Viability

**FIGURE 6:**
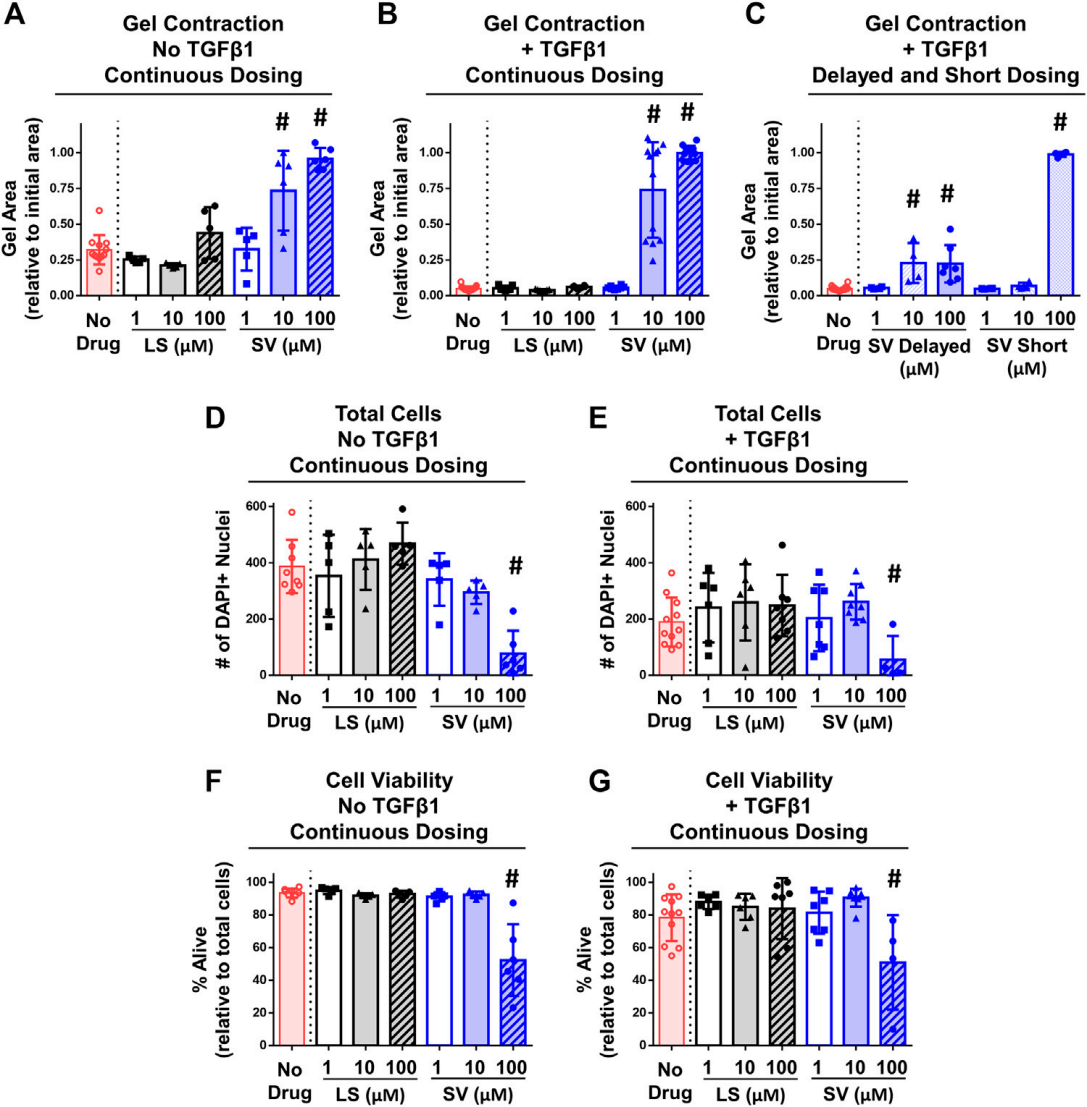
SV, but not LS, reduced fibroblasts/myofibroblasts contractility and viability *in vitro.* **(A–C)** Quantitative assessment of gel contraction area demonstrated that continuous, delayed, and short application of SV, but not LS, reduced contraction without and with TGFβ1 after 6 days. **(D–G)** Assessment of cell number and viability demonstrated that only continuous 100 µM SV treatment reduced cell number and viability without and with TGFβ1 after 6 days. Results are shown as mean ± SD; # indicates *p* < 0.05 significant difference from drug-free controls (one way-ANOVA with Dunnett’s post-hoc).

*In vitro* studies detected differences across groups in the degree of gel contraction (Figures 6A–C). On day 6 of culture, drug-free gels decreased in area from the initial size without (∼30%) and with (∼95%) TGFβ1 (Figures 6A,B). Irrespective of TGFβ1, continuous SV treatment prevented (i.e., no decreased area; Figures 6A,B) gel contraction at concentrations ≥10 µM compared to drug-free control. In contrast, no concentration of LS prevented gel contraction. Since only SV prevented gel contraction, a subset of SV- and TGFβ1-treated only gels were used to test the effect of timing and duration of SV. Similar inhibition of contraction occurred if SV was delayed and given for a short duration (Figure 6C); however, 10 µM SV no longer inhibited contraction if applied for a short duration. On day 6 after continuous drug treatment with and without TGFβ1, 100 µM SV significantly reduced cell number (∼70–80%; Figures 6D,E) and viability (∼35–45%; Figures 6F,G). In a subset of gels, there was no impact of reconstitution solvents for SV and TGFβ1 on gel contraction (Supplementary Figure S3). Overall, these results indicate that only SV could prevent gel contraction at moderate to high concentrations through modulation of NIH3T3 fibroblasts/myofibroblasts health and contractility.

## 4 Discussion

### 4.1 Overview

Currently, an unmet clinical need exists for novel therapies to prevent the development of elbow PTJC. Thus, this study tested the effects of prophylactically administered SV and LS in two preclinical models of elbow PTJC. In the rat elbow PTJC model *in vivo*, SV and LS did not prevent the onset of elbow contracture as indicated from post-mortem biomechanics (Figure 3). However, both drugs modulated capsule and cartilage biology on a region-dependent cellular and tissue level as indicated histologically (Tables 1, 2; Figures 4, 5; Supplementary Figures S1, S2). SV drug delivery appeared to decrease capsule fibrosis and cartilage damage in the joint anteriorly, yet increased damage severity in the posterior region. This observed location-dependent phenotype suggests a complex injury-drug response by which altering the anterior region of the joint with drugs might impact the biological activity in multiple tissues throughout the elbow. Neither LS dose showed any benefits and appeared to increase disease severity in both cartilage and capsule. *In vitro*, concentrations of SV, but not LS, inhibited fibroblasts/myofibroblast contractility (Figure 6). Considering the *in vitro* and *in vivo* data together suggests that both SV and LS can modulate the biological activity and tissue-level properties of capsule and cartilage. However, it appears that drug-induced cellular and tissue level changes do not necessarily translate to the functional level, at least at the single timepoint evaluated, which might have implications for future clinical and preclinical studies of elbow PTJC. Overall, these data provide a foundation of knowledge to understand better SV and LS potential as disease-modifying drugs for elbow PTJC and to guide the future optimization of dosing and delivery strategies.

### 4.2 SV and LS Induces Pleiotropic Effects on Capsule Biology *In Vivo*

Elbow PTJC is primarily driven by capsule fibrosis; hence, a study goal was to prevent capsule fibrosis post-trauma, particularly in the anterior region of the elbow since it is the location where the surgically induced injury occurs in the *in vivo* model (Lake et al., 2016; Dunham et al., 2017b). Indeed, SV given at dosing strategy 2 (60 mg/kg given 3 days/wk for 6 weeks), but not dosing strategy 1 (30 mg/kg given daily for 3 weeks), modestly reduced anterior capsule fibrosis/adhesions, although the capsule was still thick and displayed increased cellularity compared to Control (Figure 4; Table 1). At the cellular level, fibroblasts and/or myofibroblasts were histologically identified as the predominant cell type in the capsule at the timepoint evaluated (Table 1). This is in opposition to inflammatory cells (e.g., monocytes and mast cells) that are commonly implicated in knee contracture (Monument et al., 2013; David et al., 2021), yet shifts in these cell populations could occur at other timepoints post-injury or the signaling between such cells might be altered. It is unclear why SV’s effects were localized anteriorly; however, since the anterior capsule is the primary tissue of interest driving elbow contracture in this model and clinically, the increased disease severity in the posterior joint location may not be as critical. Despite modest benefits of SV, LS given at either dosing strategy 1 (20 mg/kg given daily for 3 weeks) or strategy 2 (40 mg/kg given 3 days/week for 6 weeks) failed to decrease the severity of capsule fibrosis, cartilage damage, or contracture. Given the limited timepoints following trauma and drug therapies assessed herein, it remains unknown if decreased capsule fibrosis, adhesions, and proteoglycans associated with elevated tissue thickness and cellularity indicates a delay or reduction in capsule fibrosis or tissue regeneration.

Since limited knowledge exists regarding elbow specific PTJC, as well as the use of SV and LS for PTJC in other musculoskeletal joints (e.g., knee), careful considerations should be taken when comparing this study to the literature. Nevertheless, our mixed multi-scale results somewhat align with a study by Baranowski et al., that evaluated the effects of orally administered losartan (30 mg/kg/day) and statins (in this case, atorvastatin; 15 mg/kg/day) on capsule fibrosis and joint contracture in a rat knee model of PTJC (Baranowski et al., 2019). Similar to our results, they found no functional benefit, yet both drugs still altered the capsule tissue and cellular properties. At the tissue level, atorvastatin and LS decreased and increased capsular thickness, respectively, while both drugs reduced the total cellularity and proportion of myofibroblasts at the cellular level. The efficacy discrepancy between studies could be due to the joint type, soft-tissue damage from traumatic injury, dosing strategies, timepoints evaluated, or analysis technique. Nevertheless, it appears that SV and LS can modulate the capsule’s biological activity and tissue-level properties of the elbow in a dosing strategy and anatomical location-dependent manner.

### 4.3 SV, but not LS, Prevents Fibroblasts/Myofibroblasts-Mediated Gel Contraction *In Vitro*

In this study, the gel contraction model utilizing NIH3T3 fibroblasts and TGFβ1 as a profibrotic factor (Abdalla et al., 2013; Gutiérrez et al., 2015; Negmadjanov et al., 2015) was performed to simulate *in vivo* capsule contraction (Hildebrand et al., 2014) and evaluate SV and LS’s direct effects on fibroblasts and myofibroblasts. SV, but not LS, reduced gel contraction under conditions with and without TGFβ1 in NIH3T3 fibroblasts/myofibroblasts (Figure 6). SV at higher concentration (100 µM) inhibited gel contraction mostly due to cell death, while SV at a moderate concentration (10 µM) likely altered cell contractility (assessed *via* extent of gel contraction; Figure 6). Notably, the effects of SV were transient because the gel contraction response was not altered when SV was only given for a short duration (Figure 6). Delayed application of SV (≥10 µM) led to a complete halt in gel contraction, albeit with about ∼60% contraction still occurring prior to applying SV (Figure 6). Generally, our results align with SV’s known influence on the mevalonate pathway (Stancu and Sima, 2001) and fibroblasts/myofibroblasts (i.e., reduced proliferation, viability, and contractility with ≤10 µM) (Fürst et al., 2002; Porter et al., 2004; Watts et al., 2005; Monzack et al., 2009; Burks et al., 2011; Copaja et al., 2011; Kuo et al., 2019). Further, our *in vitro* gel findings might explain/support the ability of SV to have a potential reduction in anterior capsule fibrosis by modulating fibroblasts/myofibroblasts phenotype (i.e., contractility), metabolism (e.g., deposition of fibrotic matrix), and signaling (e.g., autocrine/paracrine signaling). However, given the systemic administration of drugs in this study, SV’s impacts *in vivo* might extend beyond the direct effects on fibroblasts/myofibroblasts observed *in vitro* (e.g., modulating inflammatory cell health and signaling). Surprisingly, LS did not reduce gel contraction *in vitro* despite its known anti-fibrotic properties (Bedair et al., 2008; Burks et al., 2011; Spurney et al., 2011; Bagnato et al., 2013; Kobayashi et al., 2013; Guo et al., 2015; Huard et al., 2018; Baranowski et al., 2019; Böckmann et al., 2019). Other studies have reported similarly mixed effects, where LS inhibited gel contraction in some culture conditions (Watson et al., 1998; Benoit et al., 2008; Jia et al., 2016) yet also increased cell adhesions/invasion and proliferation (Olschewski et al., 2018). Such mixed results could be due to *in vitro* vs. *in vivo* study designs, culture conditions (e.g., collagen and cellular densities), cell type (e.g., primary vs. cell line), use of a single profibrotic factor (e.g., TGFβ1 vs. angiotensin II) and/or the presence of LS target receptor [i.e., angiotensin II type 1, which NIH3T3 fibroblasts minimally express (Heemskerk et al., 1999; De Paolis et al., 2002)]. In a subset of separate gels, angiotensin II (i.e., 10 and 100 ng/ml) was applied and had no impact on gel contraction (Supplementary Figure S3), suggesting a minimal influence of angiotensin II on NIH3T3 fibroblasts/myofibroblasts contractility. However, angiotensin II and LS might exert effects in other cellular activity of NIH3T3 fibroblasts/myofibroblasts that extend beyond gel contraction studied herein (e.g., production of growth factors/cytokines, proliferation, and adhesions/invasion). Collectively, these *in vitro* findings suggest SV, but not LS, can directly modulate cellular contractility and health of fibroblasts/myofibroblasts embedded within collagen gels, but whether SV can directly impact these cellular processes *in vivo* remains unknown.

### 4.4 SV and LS Induces Pleiotropic Effects on Cartilage Biology *In Vivo*

Elbow trauma can cause damage to other soft tissues in the elbow like cartilage; hence, this study evaluated changes to cartilage in the rat injury model of elbow post-trauma. Similar to the capsule, cartilage exhibited location- and drug-dependent changes. In both the joint’s anterior and posterior region, all cartilage changes occurred in the articular and not calcified cartilage, suggesting direct and localized effects to chondrocytes in the articular cartilage; however, this does not rule out the possibility of intracellular signaling between articular and calcified cartilage with other elbow tissues such as the capsule or subchondral bone. In both the anterior and posterior region, injury alone caused mild surface fibrillations/irregularities (Table 2; Figure 5; Supplementary Figure S2). Surface irregularities in the posterior region was unexpected since the injury model herein causes soft tissue damage to the anterior region of the joint (Lake et al., 2016; Dunham et al., 2017b); this suggests joint-wide changes not previously appreciated in this injury model of PTJC. With treatments, cartilage damage in the anterior region was slightly prevented (e.g., reduced surface irregularities/fibrillation) with both SV strategies, whereas both LS strategies caused further damage (e.g., erosions) (Table 2; Figure 5). There were notable focal regions of chondrocytes in SV treated groups that displayed enhanced clustering (Figure 5A), though this was not a widespread phenomenon across all articular cartilage. Collectively, histological observations of SV cartilage protection might be due to an overall increase in cartilage area combined with enhanced proteoglycan matrix composition in both the extracellular and pericellular matrices around chondrocytes (Figure 5). These observations in SV-treated joints suggest chondrocyte-driven attempts to repair, regeneration, and/or anabolism (Zuscik et al., 2008; Anderson et al., 2011; Goldring, 2012). However, since only one time point was evaluated post-trauma, it is unclear if this chondrocyte response would continue to be beneficial long term. Despite providing modest benefits anteriorly, both SV strategies led to articular cartilage erosions posteriorly (Table 2; Supplementary Figure S2). Although erosions developed, histomorphometric analysis revealed that the total cartilage area did not change; suggesting that the remaining non-eroded articular cartilage increases in area, as seen anteriorly, and could indicate tissue adaptation. Contrary to SV, LS was unable to provide any surface-level protection and led to areas devoid of chondrocytes (Table 2; Supplementary Figure S2).

Given the limited *a priori* knowledge of elbow-specific cartilage biology, studies from the literature utilizing SV and LS for preventing arthritis in other joints (e.g., the knee) can help interpret this study’s findings. For example, SV is thought to prevent cartilage damage by enhancing chondrogenesis (e.g., increased proteoglycan synthesis) and reducing chondrocyte production of harmful biochemical factors (e.g., inflammatory cytokines and matrix-degrading enzymes) (Yudoh and Karasawa, 2010; Aktas et al., 2011). LS appears to provide similar cartilage structure protection in the knee (Chen R. et al., 2015; Huard et al., 2018; Utsunomiya et al., 2020; Logan et al., 2021) but can also accelerate chondrocyte enlargement/hypertrophy in the growth plate (Chen S. et al., 2015). Contrary to the structural/composition benefits (e.g., prevention of fibrillations and erosions) of drug treatment on cartilage seen in these previous preclinical studies, there was no robust tissue-level improvement with most SV and LS strategies in this study. However, recent work has shown LS at higher dosages can halt cartilage repair and induce cartilage damage in healthy cartilage (Logan et al., 2021), which somewhat aligns with the deleterious effects of LS strategies in this study. Collectively, these discrepancies could be due to the dosing strategy, joint location (e.g., anterior vs. posterior), joint studied (e.g., elbow vs. knee), type of arthritis (e.g., rheumatoid arthritis vs. post-trauma osteoarthritis), and type of traumatic insult (e.g., trauma, immobilization, chemical, or combinations). Furthermore, it is unclear whether the cartilage response was directly or indirectly modulated by capsule biology, or vice versa. Nevertheless, our findings suggest that SV and LS impact the chondrocyte/cartilage injury response, but the full extent of these drugs’ impact on cartilage requires further investigations.

### 4.5 Future Work

While these data are insightful, many questions remain to be answered that will require further evaluation. Future work could increase sample sizes, modify the dosing strategies, and evaluate other drug toxicity parameters [e.g., pain, liver dysfunction, and muscle damage (MacDonald and Halleck, 2004; Schachter, 2005; Sica et al., 2005)], functional deficiencies [e.g., gait and grip strength (Reiter et al., 2019)], elbow motions [i.e., pronation and supination (Dunham et al., 2017a)], and time points post-trauma. Further, although only male rats were included in this study and previous work showed minor sex-dependent progression of PTJC (Reiter et al., 2021b), the effect of drug treatment in female rats could be different and should be considered. Since drugs were given systemically, knowledge about the pharmacokinetics and bioavailability of drugs within the elbow’s synovial space would be critical for understanding the drug mechanisms of action and optimizing and choosing alternative dosing and delivery strategies (e.g., use of nanoparticles, intra-articular injections, drinking water, or topical application). It is also important to consider that the bioavailability and effects of each drug herein and using alternative dosing and delivery strategies could also be impacted by the *in vivo* half-life of each drug [e.g., about 1–5 h in humans (MacDonald and Halleck, 2004; Schachter, 2005; Sica et al., 2005)]. Additionally, *in vivo* work should also evaluate drug concentrations and critical biomarkers of disease (e.g., TGFβ1, matrix degrading enzymes, and proinflammatory cytokines assessed *via* immunohistochemistry or other analysis techniques) in the cartilage and capsule, as well as the elbow’s synovial space and systemic blood serum. Beyond future *in vivo* work, the *in vitro* gel contraction assay would be more impactful if primary cells from the elbow capsule are used and co-culture studies are performed using other cells (e.g., macrophages and mast cells) that might communicate to fibroblasts/myofibroblasts *in vivo* (Monument et al., 2013; Hildebrand et al., 2014). Finally, understanding the underlying drug mechanism(s) driving capsule and cartilage biology changes will prove vital to fully determining the therapeutic potential of these treatment strategies.

## 5 Conclusion

In conclusion, this study demonstrated that SV, but not LS, can prevent capsule fibrosis and cartilage damage *in vivo* and cell contractility *in vitro*. In the rat elbow contracture model, orally administered SV altered histopathological evaluations at the cellular and tissue level in the capsule and cartilage but did not ultimately improve joint function at the single time point evaluated. In the gel contraction assay, SV transiently altered fibroblasts/myofibroblasts contractility. Both preclinical models demonstrated that the success of SV as a treatment for elbow PTJC will be dependent on the dosing strategy. Unfortunately, LS did not elicit a beneficial change in either the *in vivo* or *in vitro* system. Overall, results from this study support further investigation and optimization of SV dosing and delivery strategies to serve as a preventative therapy for elbow PTJC.

## Data Availability

The original contributions presented in the study are included in the article/Supplementary Material, further inquiries can be directed to the corresponding author.

## Abbreviations

PTJC, post-traumatic joint contracture; SV, simvastatin; LS, losartan; D1, dosing strategy 1 of low dose/high frequency/short duration; D2, dosing strategy 2 of high dose/low frequency/long duration; SV-D1, simvastatin dosing strategy 1; SV-D2, simvastatin dosing strategy 2; LS-D1, losartan dosing strategy 1; LS-D2, losartan dosing strategy 2; INJ, injury no drug; Control, uninjured, age-matched; TGFβ1, transforming growth factor-beta 1.
