# Supplementary material for: Pleiotropic Effects of Simvastatin and Losartan in Preclinical Models of Post-Traumatic Elbow Contracture
Source: Front Bioeng Biotechnol. 2022 Feb 21;10:803403. doi: 10.3389/fbioe.2022.803403 (PMC8899197; doi:10.3389/fbioe.2022.803403)
Supplement: Supplementary file 1 [file Table1.DOCX]

**Supplemental Table 1 Legend**

***Supplemental Table 1*** *–* Histological semi-quantitative scoring for both the capsule and cartilage is based on the histological stain (hematoxylin and eosin [H&E] vs. toluidine blue [Tol-Blue]) and whether the scoring metric is tissue or cellular level parameter. For each histological section, a semi-quantitative metric is derived from a musculoskeletal histopathologist assessment. Following assessment, the numerical scores for each elbow and group is averaged and converted into symbolic representation (-, +, ++, +++, or ++++) and used for comparisons among groups. Note: * = average # per 40x magnification in 10 hotspots; -- = metric symbol not utilized.

| **Elbow Tissue** | **Tissue or Cellar Level** | **Section Stain** | **Parameter** | **Converted Symbolic Semi-Quantitative Scores** | | | | |
| --- | --- | --- | --- | --- | --- | --- | --- | --- |
|  |  |  |  | - | + | ++ | +++ | ++++ |
| **Capsule** | **Tissue** | **H&E** | **Thickness  *(µm)*** | *<350* | *350-550* | *551-750* | *751-950* | *>950* |
|  |  | **H&E** | **Adhesions  *(% capsule adhered to bone)*** | *<10* | *10-30* | *31-60* | *>60* | *--* |
|  |  | **H&E** | **Fibrosis  *(% of capsule)*** | *<10* | *10-30* | *31-60* | *>60* | *--* |
|  |  | **Tol-Blu** | **Proteoglycan Amount  *(% tol-blue resembling cartilage)*** | *<10* | *10-30* | *31-60* | *>60* | *--* |
|  |  | **H&E** | **Vascularity*  *(# of vessels)*** | *--* | *<6* | *6-10* | *>10* | *--* |
|  | **Cellular** | **H&E** | **Cellularity*  *(# of total cells)*** | *--* | *<120* | *120-180* | *181-240* | *>240* |
|  |  | **H&E** | **Synovial Proliferation*  *(# of cells from synovial surface)*** | *<2* | *2-4* | *5-10* | *>10* | *--* |
|  |  | **H&E** | **Fibroblasts/ myofibroblasts*  *(# of elongated/spindle-like cells)*** | *--* | *<120* | *120-180* | *181-240* | *>240* |
|  |  | **Tol-Blu** | **Mast Cells*  *(# of cells)*** | *--* | *<6* | *6-10* | *>10* | *--* |
|  |  | **H&E** | **Mononuclear Inflammatory Cells* *(# of macrophages and lymphocytes)*** | *--* | *<6* | *6-10* | *>10* | *--* |
| **Cartilage** | **Tissue** | **Tol-Blu** | **Structural Damage *(any % of cartilage)*** | *normal* | *surface irregularities* | *pannus and surface irregularities* | *clefts and erosions* | *complete loss* |
|  |  | **Tol-Blu** | **Proteoglycan Matrix *(% loss)*** | *<20* | *20-39* | *40-59* | *60-80* | *>80* |
|  |  | **Tol-Blu** | **Tidemark Integrity** | *intact* | *crossed by blood vessels* | *--* | *--* | *--* |
|  | **Cellular** | **Tol-Blu** | **Cellularity** | *normal* | *diffuse hypercellularity* | *cloning* | *hypocellularity* | *-* |
