# Supplementary figures and images for: Pleiotropic Effects of Simvastatin and Losartan in Preclinical Models of Post-Traumatic Elbow Contracture
Source: Front Bioeng Biotechnol. 2022 Feb 21;10:803403. doi: 10.3389/fbioe.2022.803403 (PMC8899197; doi:10.3389/fbioe.2022.803403)

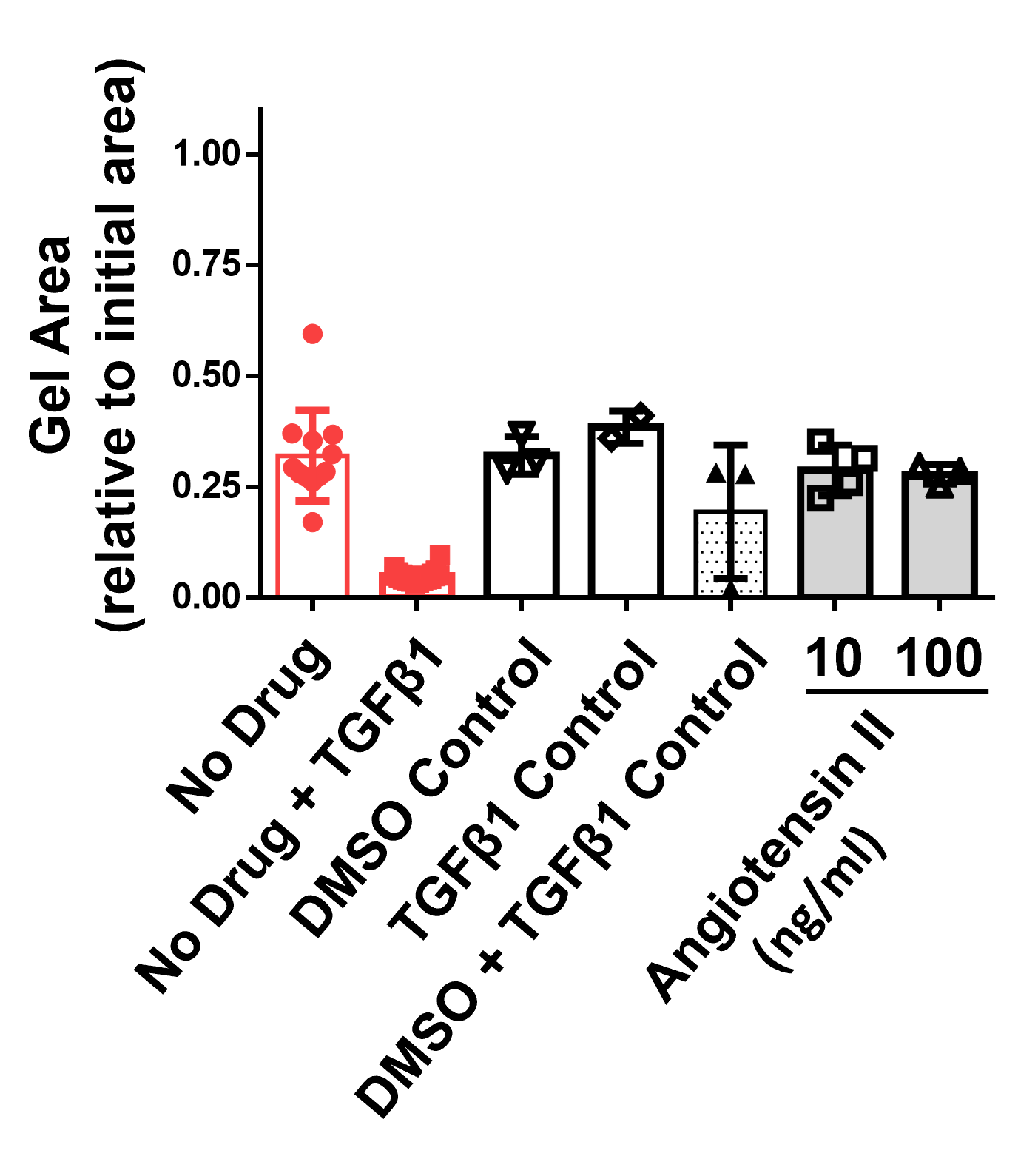

Supplement: Supplementary file 2 [file Image3.TIF]

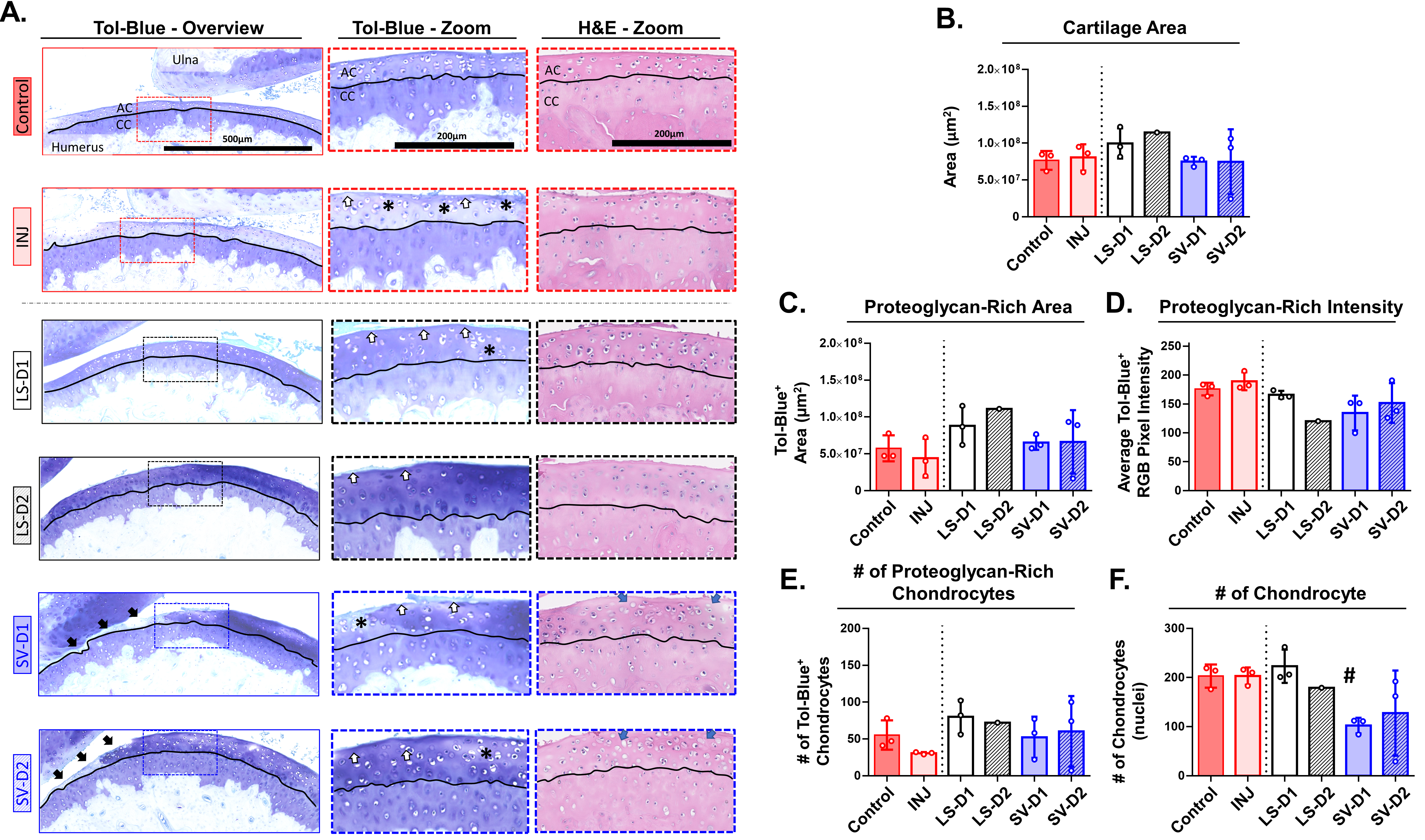

Supplement: Supplementary file 3 [file Image2.TIF]

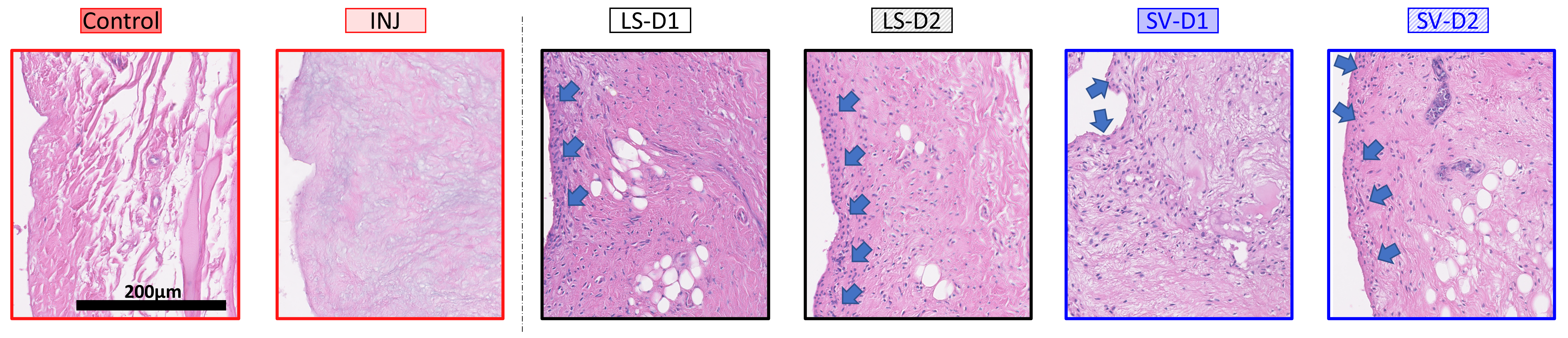

Supplement: Supplementary file 4 [file Image1.TIF]
