# Supplementary material for: Pleiotropic Effects of Simvastatin and Losartan in Preclinical Models of Post-Traumatic Elbow Contracture
Source: Front Bioeng Biotechnol. 2022 Feb 21;10:803403. doi: 10.3389/fbioe.2022.803403 (PMC8899197; doi:10.3389/fbioe.2022.803403)
Supplement: Supplementary file 5 [file DataSheet1.DOCX]

**Supplemental Figures 1 and 2 Captions**

***Supplemental Figure 1 – No drug strategy reduced capsule fibrosis posteriorly.***

Qualitative histopathology assessment on hematoxylin and eosin midsagittal sections of the posterior capsule highlights changes in the overall capsule tissue and cellular morphology. Control capsule displays loosely packed and disorganized tissue with minimal cellularity, whereas INJ caused thickened tissue and fibrosis with minimal cells. No treatment mitigated capsule fibrosis, and even displayed increased thickness, cellularity (mostly fibroblasts/myofibroblasts), synovial proliferation (arrows), and fibrosis compared to INJ and Control. Note: Control = uninjured, age-matched; INJ = injury no drug; LS-D1 = losartan dosing strategy 1; LS-D2 = losartan dosing strategy 2; SV-D1 = simvastatin dosing strategy 1; and SV-D2 = simvastatin dosing strategy 2.

***Supplemental Figure 2 – No drug strategy reduced cartilage damage posteriorly.*** (A) Qualitative histopathology assessment on Toluidine blue (Tol-Blue) and hematoxylin and eosin (H&E) midsagittal images of posterior humerus articular (AC) and calcified (CC) demonstrate minor cartilage surface-level fibrillations (white-filled arrows) and modest loss of proteoglycan (asterisks) with injury alone (INJ) compared to Control. All drug strategies showed a similar extent of cartilage damage, except SV strategies slightly worsened the damage with visible cartilage erosions on half of the articulating surface (black-filled arrows), as seen in Tol-Blue images. Note minor changes in chondrocyte morphology with the appearance of empty lacunae with SV strategies (blue-filled arrows), as seen in H&E images. (B-F) Quantitative cartilage histomorphometry at the tissue and cellular levels largely confirmed these qualitative assessments with no significant changes among groups, except a slight decrease in the number of chondrocytes. Results are shown as mean +/- standard deviation; # indicates *p* ≤ 0.05 significant difference and * indicates 0.05 < *p* ≤ 0.10 trending significance from Control (one-way ANOVA with Dunnett's post-hoc). Note: Control = uninjured, age-matched; INJ = injury no drug; LS-D1 = losartan dosing strategy 1; LS-D2 = losartan dosing strategy 2; SV-D1 = simvastatin dosing strategy 1; and SV-D2 = simvastatin dosing strategy 2.

***Supplemental Figure 3 – SV and TGFB1 reconstitution solutions, as well as Angiotensin II, did not impact gel contraction.***

Quantitative assessment of gel contraction area demonstrated that SV (i.e., 0.001% dimethyl sulfoxide [DMSO]) and TGFβ1 (i.e., 0.002 mM hydrochloric acid) reconstitution solutions, as well as Angiotensin II, did not impact gel contraction after 6 days. Results are shown as mean +/- standard deviation.
